# Membranes for the Capture and Screening of Waterborne Plutonium Based on a Novel Pu-Extractive Copolymer Additive

**DOI:** 10.3390/membranes12010003

**Published:** 2021-12-21

**Authors:** James C. Foster, Timothy A. DeVol, Scott M. Husson

**Affiliations:** 1 Department of Chemical and Biomolecular Engineering, Clemson University, 127 Earle Hall, Clemson, SC 29634, USA; jcf@clemson.edu; 2 Nuclear Environmental Engineering Sciences, Radioactive Waste Management Center, Clemson University, Clemson, SC 29625, USA; devol@clemson.edu; 3 Department of Environmental Engineering and Earth Sciences, Clemson University, 342 Computer Court, Anderson, SC 29625, USA

**Keywords:** extractive membranes, nuclear forensics, plutonium isotopic screening, alpha spectrometry, non-solvent induced phase separation

## Abstract

This contribution describes the fabrication of plutonium-adsorptive membranes by non-solvent induced phase separation. The dope solution comprised poly(vinylidene fluoride) (PVDF) and a Pu-extractive copolymer additive of PVDF-*g*-poly(ethylene glycol methacrylate phosphate) (EGMP) in dimethylformamide (DMF). The effects of casting conditions on membrane permeability were determined for PVDF membranes prepared with 10 wt% PVDF-*g*-EGMP. Direct-flow filtration and alpha spectrometry showed that membranes containing the graft copolymer could recover Pu up to 59.9 ± 3.0% from deionized water and 19.3 ± 3.5% from synthetic seawater after filtering 10 mL of 0.5 Bq/mL ^238^Pu. SEM-EDS analysis indicated that the graft copolymer was distributed evenly throughout the entire depth of the copolymer membranes, likely attributing to the tailing observed in the alpha spectra for ^238^Pu. Despite the reduction in resolution, the membranes exhibited high Pu uptake at the conditions tested, and new membrane designs that promote copolymer surface migration are expected to improve alpha spectrometry peak energy resolutions. Findings from this study also can be used to guide the development of extractive membranes for chromatographic separation of actinides from contaminated groundwater sources.

## 1. Introduction

The development of rapid analytical tools for the detection of waterborne special nuclear materials is a critical focus of nuclear forensics efforts. Trace levels of Pu exist in the environment as a result of human nuclear activity, and while weapons testing accounts for the largest source of Pu, accidental releases from nuclear power and nuclear processing facilities also have led to increased levels of Pu near those sites. The abundances of Pu isotopes in each source act as a unique signature or “fingerprint”, and the isotopic ratios can indicate where the Pu was produced and its final intended use. For example, approximately 6.1 PBq (~20 kg) of Pu was released from the disaster at Chernobyl Nuclear Power Plant in 1986, and it is estimated that the Pu consisted of 0.3% ^238^Pu, 66.9% ^239^Pu, 25.3% ^240^Pu, and 7.5% ^241^Pu by mass [1]. Unique Pu signatures also can be traced to a particular production facility, and the ^240^Pu/^239^Pu ratio can elucidate the intended use for the produced Pu. The assembly of a fission-type nuclear weapon requires a reactor design and operation to promote the growth of ^239^Pu while limiting the production of ^240^Pu. Generally, uranium target materials spend less time in the reactor and multiple batch cycles followed by chemical reprocessing are needed to produce sufficient ^239^Pu for a weapon [2]. Illicit proliferation of ^239^Pu is likely to leave trace levels of Pu in nearby waters. Due to the low solubility and complex redox chemistry of waterborne Pu, direct measurements require a multi-step approach to perform isotopic analysis. Pu isolation and pre-concentration usually cannot be performed in the field as it requires highly acidified or caustic solutions to separate Pu from a solution matrix. Therefore, an on-site analytical method that can concentrate Pu quickly to detectable limits while simultaneously screening for its isotopic abundance would be a valuable nuclear forensics tool.

Instruments such as inductively coupled mass spectrometry (ICP-MS) and thermal ionization mass spectrometry (TIMS) are capable of screening for Pu isotopes at concentrations as low as a few parts per billion; however, the size of these instruments and their respective equipment do not lend well for transport. Comparatively, an alpha spectrometer has the capability to be made into a fieldable detection system for Pu and other alpha-emitters. For high resolution spectra, samples need to be dispersed thinly over a smooth surface to reduce alpha particle energy attenuation prior to reaching the detector. For this reason, electrodeposition remains the standard method for alpha spectrometry source preparation, as it can achieve peak resolutions (reported as full-width at half maximum, FWHM) from about 15 to 55 keV for Pu isotopes [3]. Preparing an electrodeposited Pu source from a contaminated water can be challenging and time-consuming as the Pu has to be isolated through separation techniques such as coprecipitation, liquid–liquid extraction or ion-exchange chromatography. Detection limits for alpha spectrometry are on the scale of 10^−4^ Bq, meaning that high volumes of a trace-level Pu solution would need to be processed to generate the activity needed for detection [4].

To reduce the time to prepare an alpha spectrometry source, we and others have turned to developing Pu extractive membranes that can concentrate Pu to detectable limits and serve directly as the sample source. Membranes are functionalized for Pu capture either by casting a polymer-ligand film onto the membrane surface, or by utilizing a reactive surface from which a ligand layer can be grafted [5,6,7,8,9,10]. By keeping the functional layer relatively thin, high spectral resolutions can be achieved that rival electrodeposited sources. Careful consideration needs to be taken regarding the physical properties of the membrane support as the pore size and density affect overall throughput of a Pu-contaminated water source [10]. For nuclear forensics applications, the extractive membranes need to balance permeability, Pu uptake, and alpha spectrometry peak resolutions.

Pu-binding ligands are chosen based on the approach used to incorporate them in or onto a membrane, and for their ability to complex with Pu depending on its oxidation state and solution conditions. Of relevance to the current study, membranes functionalized with ethylene glycol methacrylate phosphate (EGMP) have been shown to be capable of extracting Pu over a broad pH range. The phosphate group (with pK_a_ values of 1.6 and 6.6) can interact with Pu through ion-exchange or by chemical coordination. The dual functionality of EGMP was demonstrated in a study by Vasudevan et al., where polypropylene membranes grafted with EGMP recovered greater than 90% of Pu(IV) from solutions ranging from 0 to 4 M nitric acid [11]. Other studies have shown that membranes containing EGMP can serve directly as an alpha spectrometry sample source for actinides. Duval et al. developed a method to graft EGMP from polyethersulfone membranes for the isotopic screening of waterborne uranium. After filtering solutions spiked with ^233^U, the modified membranes achieved ^233^U peak resolutions ranging from 17 to 33 keV FWHM [12]. Furthermore, the peak resolutions achieved by the membranes were sharp enough to distinguish ^238^U (4.198 MeV) from ^234^U (4.775 MeV) after filtering a spiked groundwater source at pH 6.

In our previous study [13], extractive thin-film composite (e-TFC) membranes were developed for the rapid capture and isotopic screening of Pu in water. The e-TFC membranes were made by spin coating a thin copolymer film consisting of 5% (*w*/*w*) EGMP and 4-methylstyrene (a.k.a. vinyltoluene, VT) onto a poly(vinylidene fluoride) (PVDF) ultrafiltration membrane. Direct flow filtration studies indicated that the e-TFC membranes recovered up to 10.2 ± 4.2% of ^242^Pu after filtering 10 mL of a 4.53 Bq/mL ^242^Pu solution at pH 6.8, and the 16 h alpha spectra exhibited average energy resolutions of 71.7 ± 8.7 keV for the ^242^Pu peak. While spin coating provided a simple and effective way to cast a Pu-selective polymer onto a membrane, the addition of the poly(EGMP-*co*-VT) layer greatly reduced the overall permeability of the membrane. At trace-level Pu concentrations (10^−12^ to 10^−10^ M), the reduced permeability of the e-TFC membranes poses concern as the membranes need to be able to process large volumes of solution rapidly to reach alpha spectrometry detection limits.

The aim of this study was to develop a Pu-extractive membrane with high permeability for rapid concentration and alpha spectroscopy analysis of trace-level Pu from water. For high alpha spectra energy resolution, the extractive functional layer should be thin and able to sequester Pu onto the surface of the membrane. The method for membrane fabrication was inspired by the approach used by Hester et al. [14] to develop fouling-resistant graft copolymer membranes. They demonstrated that hydrophilic poly(oxyethylene methacrylate) (POEM) and poly(methacrylic acid) (PMAA) could be grafted directly by atom transfer radical polymerization (ATRP) from the secondary fluorinated sites in PVDF. This created amphiphilic copolymers consisting of a hydrophobic PVDF anchor with a hydrophilic POEM or PMAA comb shell. PVDF membranes were prepared by nonsolvent induced phase separation (NIPS) using dope solutions containing 5–10 wt% PVDF-*g*-POEM. It was observed that the amphiphilic nature of the copolymer would lead it to segregate to the membrane surface upon formation. In membranes containing 10 wt% PVDF-*g*-POEM, the surface migration greatly improved the wetting of the membrane and reduced protein adsorption over seven-fold compared to PVDF membranes. This study demonstrated a simple approach to casting functional membranes without the need for post-modification steps. Here, we used ATRP to graft EGMP from PVDF. We theorized that the addition of this Pu-selective PVDF-*g*-EGMP copolymer in the dope solution during membrane formation by NIPS would yield a high permeability, Pu-extractive membrane. We theorized further that, given the hydrophilicity of EGMP, the graft copolymer would migrate to the surface of the membrane where it would concentrate Pu during filtration and enable high spectral resolution when the membrane is screened in an alpha spectrometer.

## 2. Materials and Methods

### 2.1. Materials

The following chemicals were purchased from MilliporeSigma (St. Louis, MO, USA): copper(I) chloride (CuCl, 97%); dimethylformamide (DMF, 99.8%); inhibitor remover (aluminum oxide, activated); methanol (95%); n-methyl-2-pyrrolidone (NMP, 99.5%); phosphoric acid 2-hydroxyethyl methacrylate ester (further denoted as ethylene glycol methacrylate phosphate, EGMP, 90%); PVDF_250K_ (M_w_ ~250,000 g/mol); PVDF_534K_ (M_w_ ~534,000 g/mol); and tris(2-pyridyl methyl) amine (TPMA, 98%). Chemicals purchased from Fisher Scientific (Waltham, MA, USA) were nitric acid (HNO_3_, 65% *w*/*w*); petroleum ether (ACS reagent, 35–60 °C boiling range); and sodium hydroxide (NaOH, 97%). Instant Ocean Synthetic Sea Salt was purchased from Carolina Biological Supply Company (Burlington, NC, USA). Nitrogen gas and dry air was from Airgas (Radnor, PA, USA). Type-1 deionized (DI) water was produced using a Millipore Milli-Q Reference water purification system (Darmstadt, Germany).

^242^Pu solutions were prepared from a 147 Bq/mL ^242^Pu(VI) (PuO_2_^2+^) stock in 1 mM HNO_3_ provided by Dr. Brian Powell (Clemson University, Department of Environmental Engineering and Earth Sciences). Low-activity solutions were diluted from the stock with DI water, followed by conditioning with 0.1 and 1 M aliquots of HNO_3_ and NaOH to achieve circumneutral pH conditions immediately prior to use. Table S1 in Supplementary Materials reports mass fractions of the Pu isotopes in the stock solution.

For the direct flow filtration study, ^238^Pu solutions were prepared by dilution in DI water and synthetic seawater from a NIST traceable ^238^Pu stock (Eckert & Ziegler, Valencia, CA, USA) with an activity of 2464 Bq/mL in 0.1 M HNO_3_. The dilutions were conditioned with 0.1 and 1 M aliquots of HNO_3_ and NaOH to adjust the pH to circumneutral conditions. The solutions were set aside for 3 d to allow for equilibrium between Pu oxidation states. Synthetic seawater was prepared using Instant Ocean Synthetic Sea Salt to replicate typical seawater conditions. Table S2 in Supplementary Materials provides the composition of the solution. Pu oxidation states of the ^238^Pu dilutions were determined through a solvent extraction method outlined by Powell et al. [15].

### 2.2. Synthesis of PVDF-g-EGMP Copolymer

Prior to copolymer synthesis, monomethyl ether hydroquinone was removed from the EGMP by combining 1 g EGMP and 20 mg inhibitor remover and stirring for at least 1 h. A Cu-TPMA solution was prepared by dissolving 40 mg CuCl and 180 mg TPMA in 5 mL NMP under an inert N_2_ atmosphere in a glovebox (MBRAUN, Garching, Germany).

An EGMP solution was prepared in a Schlenk flask by dissolving 15 g EGMP in 20 mL NMP. In a separate Schlenk flask, 5 g PVDF_250K_ was dissolved in 30 mL NMP. Dissolved air was removed from the EGMP and PVDF_250K_ solutions through a freeze-pump-thaw procedure. First, the flasks were sealed and placed in a liquid nitrogen bath until the contents were frozen solid. A vacuum line was attached, and the flasks were submerged in a 75 °C water bath while under vacuum to thaw. This process was repeated for at least three cycles, or until visible air bubbles were no longer formed during the thawing step. The EGMP and PVDF_250K_ solutions were left sealed under vacuum until further use.

ATRP grafting of EGMP from PVDF was performed in the N_2_ glove box where the Cu-TPMA solution was stored. The prepared EGMP and PVDF_250K_ solutions were combined in a reaction vessel and heated to 90 °C stirring by a magnetic stir bar at 300 RPM. Once the set temperature was reached, the Cu-TPMA solution was added to the reaction mixture, and the ATRP reaction proceeded for 24 h. Afterwards, the reaction vessel was removed from the glove box. PVDF-*g*-EGMP was precipitated from solution with a mixture of 50 mL methanol and 100 mL petroleum ether. To remove unreacted EGMP monomer, the precipitated PVDF-*g*-EGMP was dissolved again in NMP, followed by precipitation with the methanol/petroleum ether mixture. The copolymer was dried for at least 24 h in a fume hood at room temperature. Characterization of PVDF-*g*-EGMP was done by attenuated total reflectance Fourier-transform infrared spectroscopy (ATR-FTIR) on a PerkinElmer Spectrum II equipped with a diamond crystal. Spectra were generated from 32 scans at a resolution of 8 cm^−1^ using PerkinElmer Spectrum 10 software.

### 2.3. Screening of PVDF-g-EGMP Copolymer for ^242^Pu Uptake

Pu-uptake measurements were performed with PVDF-*g*-EGMP to quantify Pu uptake from solutions at neutral pH conditions. A ^242^Pu(VI) solution was diluted from the ^242^Pu stock to produce a solution with a final activity of 2.67 Bq/mL and pH 6.92. One hundred milligrams of the PVDF-*g*-EGMP copolymer was placed in a 1.5 mL vial with 1 mL of the ^242^Pu solution. The sample was stirred for 24 h on a Stuart SB2 rotator (Staffordshire, UK) set to 20 RPM. Afterwards, 0.5 mL of solution from each sample was removed and pipetted onto a 30 mm diameter steel planchette. Once planchettes were dry, they were placed 5 mm from the detector face in an alpha spectrometer and counted for 5 h. A control study was performed by repeating the study with PVDF_534K_ in-lieu of the grafted copolymer, and Pu sorption to the vials was accounted for by performing the experiment with empty vials. All sample trials were performed in triplicate measurements to determine average values and standard deviations for ^242^Pu uptake.

### 2.4. Casting PVDF Membranes with PVDF-g-EGMP

Grafted copolymer membranes were cast from dope solutions using the formulations shown in Table 1, designed to produce membranes composed of approximately 10 wt% PVDF-*g*-EGMP. Dope solutions were prepared by dissolving PVDF_534K_ and PVDF-*g*-EGMP in DMF for 24 h under stirring, or until the polymer was fully dissolved. To study the effects of permeability on Pu uptake and alpha spectra peak resolution, glycerol was added to the dope solutions at 2 wt% to increase the porosity of the membranes.

Membrane formation was achieved through NIPS. Prior to membrane casting, the polymer dope solutions were vacuum degassed at 75 kPa to remove any dissolved air from solution. The solutions were cast onto a clean glass plate and drawn using a doctor blade (Gardco, Pompano Beach, FL, USA) set to a height of 0.18 mm. Five seconds after drawing, the glass plate was immersed in a DI water bath for at least 2 min, resulting in the complete coagulation of the membrane onto the glass plate. Afterwards, the membranes were removed from the bath and stored in fresh DI water until further use.

### 2.5. Permeability Studies

Grafted copolymer membranes were cut to 45 mm diameter coupons and placed in a Sterlitech HP4750 flow cell (Kent, WA, USA). The cell was filled with DI water and sealed. Prior to measurements, the flow cell was pressurized to 4.14 barg for 15 min to condition the membrane prior to taking measurements. Afterwards, the operating pressure was reduced to 0.69 barg. Flux measurements were taken at four setpoints as the pressure was increased from 1.38 to 3.45 barg. At each pressure setpoint, filtrate mass and flow time were recorded to determine water flux and permeability. After the first pressure cycle, cell pressure was reduced to 0.69 barg, and measurements were taken over a second pressure ramp to observe whether hysteresis occurred. For each membrane casting condition studied, average permeability values and uncertainty were calculated from five membrane samples.

### 2.6. SEM Analysis of Membrane Morphologies

Scanning electron microscopy imaging with electron dispersive spectroscopy (SEM-EDS) was performed on the copolymer membranes to visualize morphology and locate where PVDF-*g*-EGMP copolymer resides within the membrane structure. Prior to measurements, membrane samples were washed sequentially for 1 h increments in water/ethanol solutions consisting of 25, 50, and 75 wt% ethanol. After drying from the 75 wt% ethanol solution, the samples were mounted to an aluminum holder with carbon tape. Samples were prepared so that the topside and cross-section morphologies could be analyzed. To analyze the membrane cross-section, samples were submerged in liquid nitrogen, freeze fractured, and mounted to the aluminum holder with the fractured edge exposed.

Samples for topside surface imaging were coated with platinum for 40 min using an Anatech Hummer 6.5 sputter coater (Sparks, NV, USA). Micrographs were taken of the membrane surfaces using a Hitachi Regulus 8230 SEM (Tokyo, Japan) at an accelerating voltage of 2 kV. For the cross-sectional samples, platinum coating was not required; instead, the samples were measured on a Hitachi SU5000 SEM equipped with an Oxford Instruments X-Max 80 silicon drift detector for EDS (High Wycombe, UK). The samples were screened at an accelerating voltage of 10 kV, and chemical imaging of the cross-section was performed using AztectLive v3.3 software (Oxford Instruments, High Wycombe, UK). Pore size diameter measurements were averaged over twenty pores using ImageJ image analysis software, freely available online from the National Institutes of Health (Bethesda, MD, USA).

### 2.7. Alpha Spectrometry Measurements

Alpha spectrometry was performed using an Ortec 7401 alpha spectrometer (Oak Ridge, TN, USA) equipped with a 450 mm^2^ passivated ion silicon detector (PIPS). Peak energy calibration was performed with a 30 mm diameter Eckert & Ziegler electrodeposited source containing known quantities of ^234^U, ^238^U, ^239^Pu, and ^241^Am. The calibration source was placed 5 mm from the PIPS detector and counted for 1 h.

Absolute detector efficiency was determined for the 45 mm diameter membranes by spotting 5 Bq of the stock ^238^Pu solution onto the surface of a PVDF membrane. The droplets were carefully smeared across the membrane surface using a pipette tip. Once dry, the membrane was fixed into a custom acrylic holder and placed 5 mm away from the PIPs detector to be counted for 1 h.

### 2.8. Direct Flow Filtration of ^238^Pu Solutions through PVDF-g-EGMP Membranes

Membranes cast with 10 wt% PVDF-*g*-EGMP were cut into 45 mm diameter coupons and placed in a Amicon ultrafiltration cell (EMD Millipore, Burlington, MA, USA). Two ^238^Pu solutions were prepared for the study: a spiked DI water solution with an activity of 0.48 ± 0.01 Bq/mL and pH 6.61, and a synthetic seawater solution with an activity of 0.51 ± 0.01 Bq/mL and pH 6.58. Filtration and alpha spectrometry procedures were the same for DI water and synthetic seawater trials. The Amicon flow cell was filled with 10 mL of the prepared ^238^Pu solution. The cell was sealed and pressurized to 0.69 barg using a dry air cylinder to start filtration. Once all of the ^238^Pu solution was filtered, the cell was refilled with 10 mL DI water and pressurized to 0.69 barg to perform a wash cycle to remove Pu from the membrane pores. The membrane was removed once the cell was completely empty of its contents and depressurized. Membrane samples were dried for at least 1 h before alpha spectrometry measurements. Alpha spectrometry was performed by mounting the membranes in a custom acrylic holder placed 5 mm from the detector face, followed by counting for 5 h.

## 3. Results

### 3.1. PVDF-g-EGMP Synthesis and Characterization

**Figure 1:**
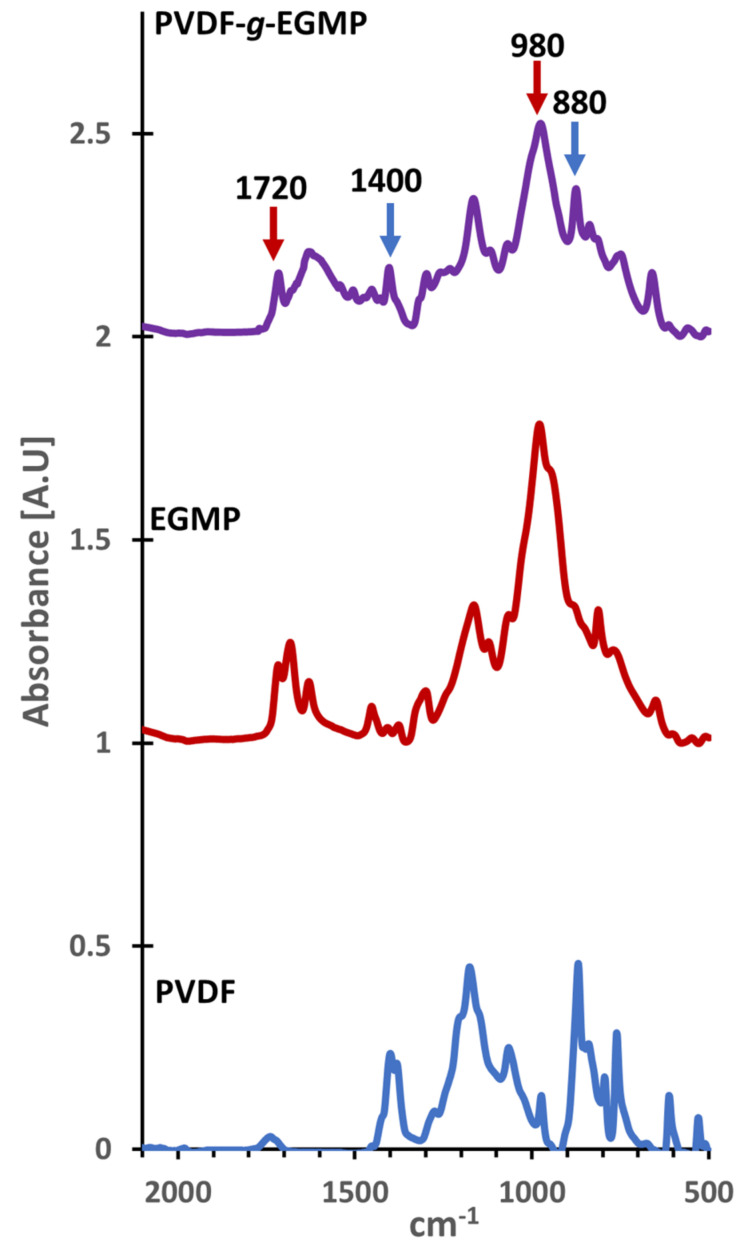
Attenuated-total reflectance Fourier-transform infraredspectra of the PVDF-*g*-EGMP copolymer, EGMP monomer, and PVDF used in atom transfer radical polymerization (ATRP)grafting.

FTIR analysis was performed on the PVDF-*g*-EGMP copolymer to determine if it exhibited characteristic peaks of EGMP. Figure 1 shows FTIR spectra for the graft copolymer, along with EGMP and PVDF precursors. Distinct peaks for the two precursors were observed in the graft copolymer, indicating successful grafting of EGMP from the PVDF macroinitiator. Characteristic absorbance peaks for EGMP were noted at 1720 and 980 cm^−1^ assigned to C=O and P=O stretching, while peaks at 1400 and 880 cm^−1^ correspond to vibrational bands observed in semi-crystalline PVDF [16,17].

### 3.2. Permeability Measurements of PVDF-g-EGMP Membranes

Direct-flow water permeability measurements were carried out for the 10 wt% PVDF-*g*-EGMP copolymer membranes. Casting conditions were varied to determine the effects on membrane permeabilities. The copolymer membranes were cast (1) with or without vacuum degassing of the polymer solution prior to casting; (2) with or without the addition of 2 wt% glycerol; and (3) by waiting a short (5 s) or long (30 s) period between casting and submerging into the coagulation bath. Casting conditions are summarized in Table S3, and the resulting water flux data for each membrane are given in Figure S1. The linear relationship between water flux and transmembrane pressure is given by Equation (1), where *J* represents water flux (in L/m^2^/h, further denoted as LMH), *A* is the permeability coefficient (LMH/bar), and *ΔP* is the transmembrane pressure (bar).

(1) J=AΔP

**Figure 2:**
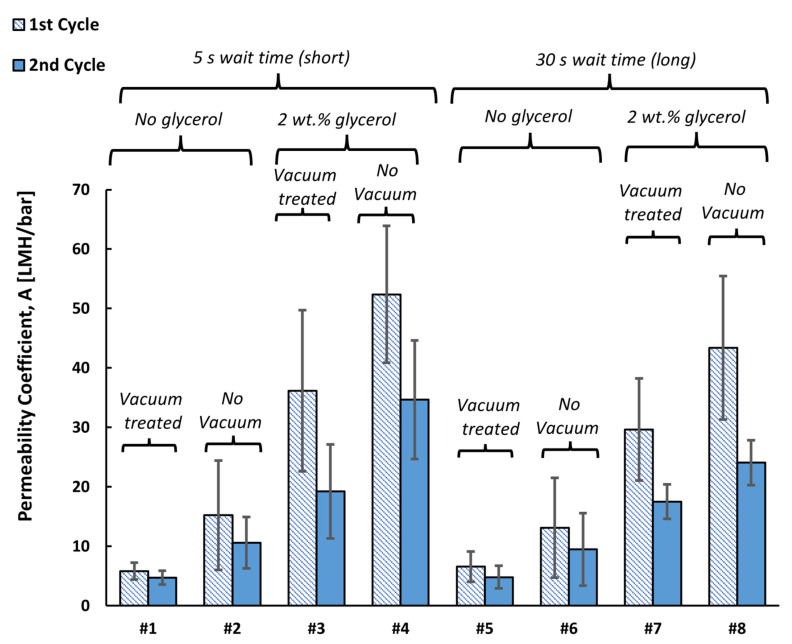
Average permeability coefficients of 10% PVDF-*g*-EGMP membranes prepared with various casting conditions. Error bars represent ±1σ for five separate sample measurements.

Figure 2 shows the average permeability coefficients for each membrane, along with the conditions used to prepare the membranes. The permeability of the membranes increased with the incorporation of glycerol and when the copolymer dope solution was not degassed by vacuum prior to casting. The increase in permeability for non-degassed samples was likely due to dissolved air present in the membrane casting solutions, potentially leading to the formation of voids during the coagulation process. Degassing is often a critical step in phase inversion processes as it prevents the formation of microbubbles as the polymer begins to solidify [18].

To determine whether the short or long wait times affected the overall permeability of the copolymer membranes, comparisons were made between treatments using a two-tailed separate-variance (Welch’s) *t*-test. At an α level of 0.05, it was observed for the two wait times that differences in permeability were significant only in samples with the highest permeability coefficients (treatments 4 and 8). By increasing the wait time, it is possible that the polymer film will slowly begin to solidify at the surface. In a study on modified PVDF membranes by Feng et al. [19], it was observed that long wait times resulted in membranes with a denser surface layer, fewer pores per area, and a finger-like cross-sectional structure. The structure results from the increased polymer concentration at the surface as the solvent evaporates, thereby inhibiting the rate at which the polymer can coagulate and the diffusion of the solvent out of the polymer.

The inclusion of PVDF-*g*-EGMP within the membrane led to a significant increase in water permeability. When PVDF membranes were cast under the same conditions without glycerol, observable permeate flow was achieved only at pressures above 5 barg. Due to the negatively charged phosphate group in PVDF-*g*-EGMP, the copolymer membranes share similar structure to other ion-conducting polymers used to create polymer electrolyte membranes (PEMs) [20]. Prior studies on PEMs have observed an increase in wetting in membranes with long alkyl phosphonic chains [21]. It is likely that the phosphate group in PVDF-*g*-EGMP aids in the wetting of the pores in the copolymer membrane and increases water permeation.

### 3.3. ^242^Pu Uptake by PVDF-g-EGMP Copolymer

^242^Pu batch uptake studies were performed to evaluate the effectiveness of the PVDF-*g*-EGMP copolymer to extract Pu(VI) from water at pH 6.92 after 24 h of contact. ^242^Pu uptake was calculated by Equation (2).

(2) P 242u uptake=Ablank−ApolymerAblank×100%

**Figure 3:**
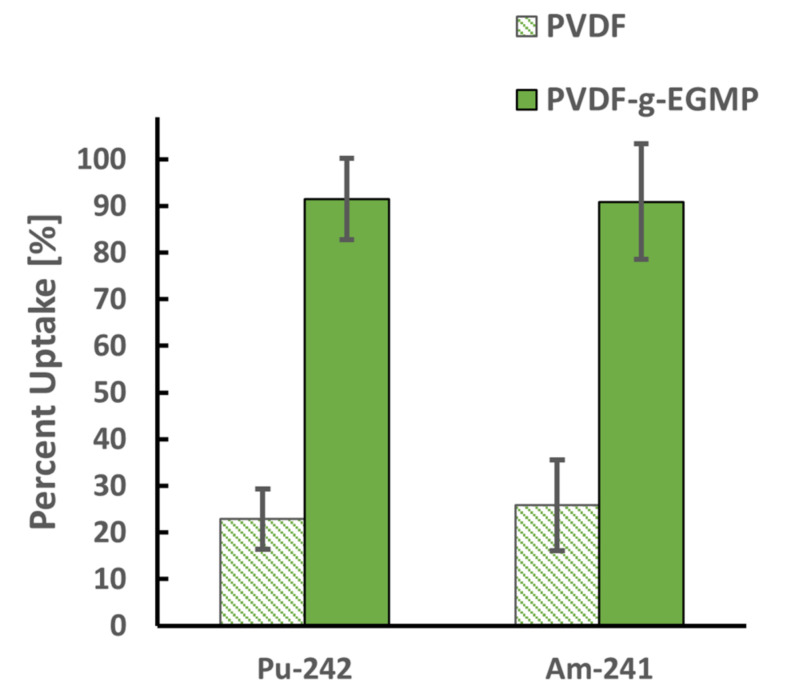
^242^Pu and ^241^Am uptake by PVDF and PVDF-*g*-EGMP samples from a solution containing 2.67 Bq/mL ^242^Pu at pH 6.92. Measurements were taken after 24 h contact with stirring. Error bars represent ±1σ for triplicate measurements.

^242^Pu uptake percentages of the PVDF_534K_ and PVDF-*g*-EGMP was determined by measuring the final ^242^Pu activity (Bq) of solution in the empty (blank) vials (*A_blank_*) and of the vials containing polymer (*A_polymer_*). Due to the age of the ^242^Pu source, ^241^Am (daughter product of ^241^Pu decay) was present in the ^242^Pu solution and accounts for approximately 20% of the gross alpha activity in the solution. Activity measurements were performed on the alpha spectrometer to distinguish ^242^Pu activity from ^241^Am, allowing for Equation (2) to be used to determine ^241^Am uptake as well. Figure 3 shows the ^242^Pu and ^241^Am uptake values for the PVDF_534K_ and PVDF-*g*-EGMP. Compared to PVDF_534K_, uptakes of ^242^Pu and ^241^Am were significantly higher for PVDF-*g*-EGMP, resulting in near-complete uptake of the two actinides from solution under the test conditions. EGMP can bind Pu through ion exchange coordination depending on the solution conditions and pH. In acidic conditions, studies by Chappa et al. have shown that EGMP can coordinate with Pu through the phosphoryl and carbonyl oxygens as a bidentate ligand [22]. At higher pH, the phosphoric acid group becomes deprotonated (pK_a1_ and pK_a2_ values of 1.6 and 6.6) and is capable of cation exchange with Pu. The copolymer performed well for the uptake of Pu at a neutral pH due to EGMP acting as an ion-exchange site [23]. At the conditions tested, PVDF-*g*-EGMP appears to be non-selective between Pu(VI) and Am(III), but further sorption experiments will need to be done to evaluate whether the copolymer is selective for one actinide over the other.

### 3.4. Direct Flow Filtration and Alpha Spectrometry Measurements with 10% PVDF-g-EGMP Membranes

Membranes composed of 10 wt% PVDF-*g*-EGMP were examined for their ability to extract Pu from water by direct flow filtration and to determine if Pu-loaded membranes can serve as samples for isotopic screening via alpha spectrometry following filtration. Three membranes were prepared for the study. Following the formulations presented in Table 1, two sets of PVDF-*g*-EGMP membranes were cast with 0 and 2 wt% glycerol to create membranes with low and high permeabilities. A third set of membranes composed of 100% PVDF and cast with 2 wt% glycerol was used as a control for the study. ^238^Pu was chosen as the isotope as its higher specific activity allows for substantially lower mass concentrations of Pu to be screened. ^238^Pu-spiked DI water and synthetic seawater (further denoted as seawater) solutions were adjusted to a neutral pH and aged for 3 d to establish equilibrium between the Pu oxidation states. Prior to testing, the spiked DI water solution measured an activity of 0.48 ± 0.01 Bq/mL (~3.2 × 10^−12^ M) at pH 6.61, and redox analysis indicated a composition of 34.2% Pu(IV) and 65.8% Pu(V). The activity of the seawater solution was measured to be 0.51 ± 0.01 Bq/mL (~3.4 × 10^−12^ M) at pH 6.58 and was composed of 36.7% Pu(IV) and 63.3% Pu(V).

**Figure 4:**
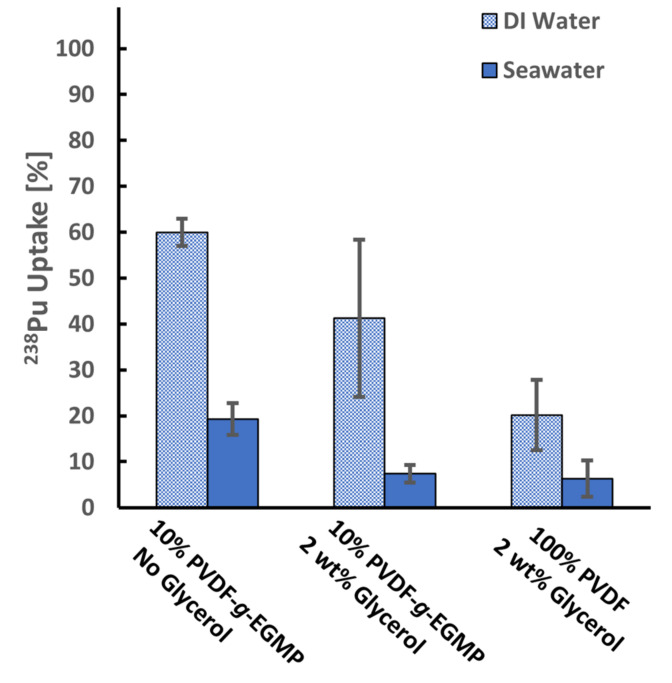
^238^Pu uptake of 100% PVDF and 10% PVDF-*g*-EGMP membranes after filtration of 10 mL of spiked deionized (DI) water (pH 6.61, 0.48 Bq/mL) and synthetic seawater (pH 6.58, 0.51 Bq/mL). Error bars represent ±1σ for three separate membranes.

Figure 4 shows ^238^Pu uptake percentages for the PVDF and PVDF-*g*-EGMP membranes. Highest ^238^Pu uptake percentages were observed for the PVDF-*g*-EGMP membranes cast without glycerol, with an average of 59.9 ± 3.0% in DI water and 19.3 ± 3.5% in seawater. ^238^Pu uptake from DI water was also high for the PVDF-*g*-EGMP membranes with glycerol at 41.3 ± 17.1%. Despite the lower ^238^Pu uptake observed for the PVDF-*g*-EGMP membranes cast with 2 wt% glycerol, the increased permeability drastically reduced the time required for filtration. At the same operating pressure, the copolymer membranes achieved flowrates of 1.57 ± 0.80 mL/min and 17.99 ± 4.74 mL/min without and with the addition of glycerol.

The overall decrease in Pu uptake in seawater likely resulted from two compounding factors: the presence of other ions that can compete for ion-exchange sites on EGMP and a change in the near-surface charge (zeta potential) of the membranes due to charge screening. The presence of other cations at high concentration potentially can reduce uptake of Pu and other actinides by EGMP. In a recent study by Suresh and Duval [24], adsorption isotherm experiments for uranyl and prominent seawater cations were performed with membranes consisting of an EGMP-based graft copolymer. Values for the equilibrium adsorption capacities and the Langmuir constants were reported for the cations after fitting to a single-site Langmuir model. While the membranes exhibited a high sorption capacity for UO_2_^2+^, other divalent cations (Ca^2+^ and Mg^2+^), Fe^3+^ and VO_2_^+^ had similar or higher Langmuir constants. The study demonstrated that EGMP binds these ions, which reduces uptake of target actinides. Coulombic interactions between Pu ions and the membrane surface also are impacted by the high ionic strength of the seawater solution. At a neutral pH, pristine PVDF membranes have a negatively charged surface and exhibit a zeta potential of approximately −40 mV [25]. The negative surface charge attracts Pu cations towards the surface, resulting in the appreciable Pu uptake measured for pristine membranes. However, studies have shown that the zeta potential in PVDF membranes approaches a net zero charge as salt concentrations are increased. The reduction in the electrochemical boundary layer likely reduces the attraction of Pu ions to the surface of the membrane [26].

**Figure 5:**
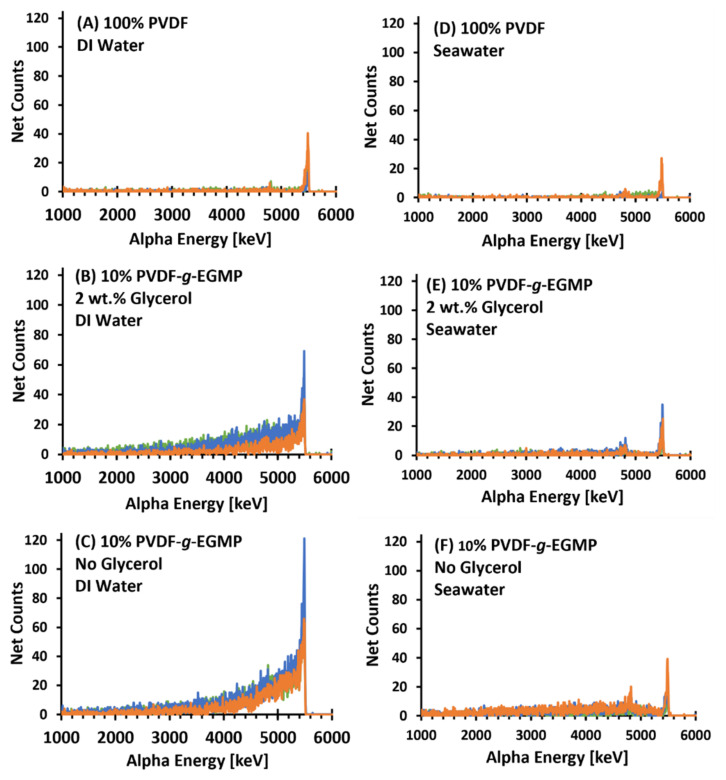
Five-hour alpha spectra of 100% PVDF, 10 wt% PVDF-*g*-EGMP (2 wt% glycerol), and 10 wt% PVDF-*g*-EGMP (no glycerol) membranes after direct filtration of Pu-bearing solutions. Measurements were taken after filtering 10 mL of DI water (Spectra **A**–**C**, 0.48 Bq/mL ^238^Pu, pH 6.61) and synthetic seawater (Spectra **D**–**F**, 0.51 Bq/mL ^238^Pu, pH 6.58).

Isotopic analysis of the membranes was performed after filtration, and Figure 5 shows the resulting alpha spectra for the DI water and seawater trials collected over 5 h. Due to retention of ^238^Pu on the negatively charged surface, the 100% PVDF membranes exhibit small but sharp alpha energy peaks for the radioisotope. In the DI water trials, spectra for membranes composed of 10 wt% PVDF-*g*-EGMP had higher ^238^Pu counts compared to the PVDF membranes. Despite differences in Pu uptake, the overall shape of the ^238^Pu photopeak was similar between PVDF-*g*-EGMP membranes cast without and with glycerol. Average peak energy resolutions were 48 keV FWHM for the copolymer membranes cast without glycerol (Figure 5C) and 32 keV FWHM for membranes cast with glycerol (Figure 5B). The PVDF-*g*-EGMP membranes exhibited broad tailing in the ^238^Pu photopeak that accounted for approximately 83% (no glycerol) and 85% (2 wt% glycerol) of the total ^238^Pu activity measured. Despite the lower ^238^Pu uptake observed in the seawater trials, slight tailing was also observed in the PVDF-*g*-EGMP samples without glycerol (Figure 5F).

**Figure 6:**
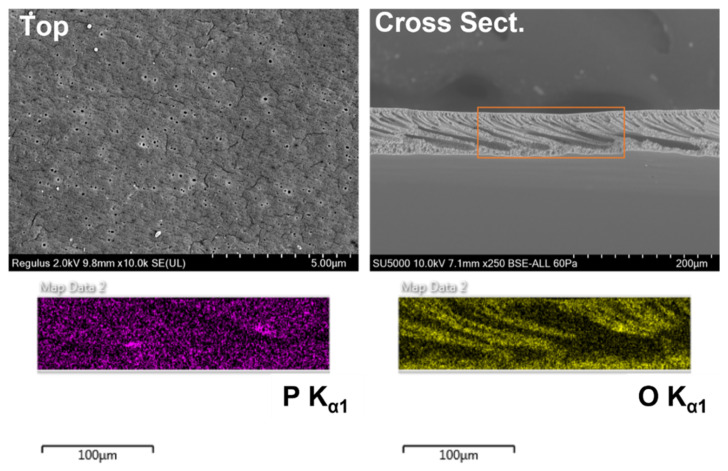
SEM-EDS images of 10 wt% PVDF-*g*-EGMP membranes cast without glycerol. Phosphorus (purple) and oxygen (yellow) distributions are shown for the region highlighted in the cross-sectional image. Average pore diameter was measured to be 0.098 ± 0.027 µm.

**Figure 7:**
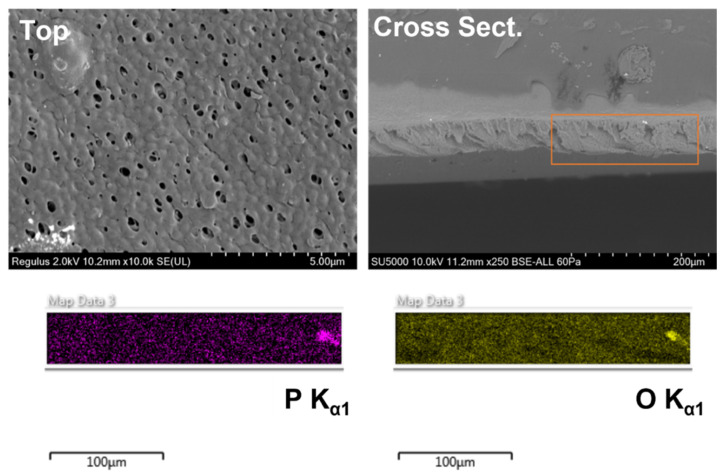
SEM-EDS images of 10% PVDF-*g*-EGMP membranes cast with 2 wt% glycerol. Phosphorus (purple) and oxygen (yellow) distributions are shown for the region highlighted in the cross-sectional image. Average pore diameter was measured to be 0.338 ± 0.066 µm.

The overall shape of the spectra seen in the copolymer membranes indicates that Pu sorption occurs on both the surface and pore walls of the membrane, resulting in a composite spectrum [27]. While Pu sorption to the surface creates a sharp photopeak, sorption to the pore walls results in broad tailing as the emitted alpha particles collide with the surrounding polymer before reaching the detector. We hypothesized that the amphiphilic structure of PVDF-*g*-EGMP would cause it to migrate to the surface during the casting of the membrane; however, if Pu sorption is occurring within the pore walls, it is likely that the graft copolymer is distributed throughout the depth of the membrane. To test this theory, SEM-EDS micrographs were taken of the membrane cross sections to determine the distribution of the PVDF-*g*-EGMP copolymer. SEM-EDS images for the PVDF-*g*-EGMP membranes are shown in Figure 6 (no glycerol) and Figure 7 (2 wt% glycerol). Locating PVDF-*g*-EGMP within the cross section was done by measuring P K_α1_ (2.013 KeV) and O K_α1_ (0.525 KeV) X-rays emitted from the phosphate and carboxylate ester groups found in EGMP. Images from the SEM-EDS analysis show that PVDF-*g*-EGMP appears to be distributed evenly throughout both membrane samples. The pore structure of the membrane can be observed clearly in the P K_α1_ and O K_α1_ micrographs for the membranes cast without glycerol, indicating that the graft copolymer lines the pore walls.

While the even distribution of PVDF-*g*-EGMP throughout the membrane aids in the recovery of Pu overall, it impairs alpha spectrometry analysis; the peak tailing reduces its ability for high-resolution isotopic screening. However, changes to the synthesis of the PVDF-*g*-EGMP, as well as how the membrane is cast, can further promote surface migration of the copolymer within the membrane. Similar studies have been performed to promote the migration of amphiphilic copolymers to create a hydrophilic, fouling-resistant membrane surface [28]. Hester et al. [29] screened novel casting methods to promote surface migration of poly(MMA-*r*-POEM) in PVDF membranes. Surface concentrations of poly(MMA-*r*-POEM) were the highest when the membranes were coagulated in a hot water bath (~90 °C) followed by 24 h annealing at 90 °C, and it was speculated that the annealed membranes reached a maximum limit of copolymer surface coverage. In a later study, Hester and Mayes observed an increased surface concentration of poly(MMA-*r*-POEM) in a PVDF membrane when the molecular weight of the amphiphilic copolymer was increased [30]. Higher molecular weights of PVDF-*g*-EGMP can be achieved by allowing the ATRP reaction to proceed for longer durations, increasing the length of poly(EGMP) chains grafted from the PVDF anchor. This approach potentially may improve the resulting alpha spectra, as the higher ratio of EGMP to PVDF may promote segregation of the copolymer to the membrane surface during membrane formation.

From a broader perspective, the functional membranes presented in this study could serve as extractive membrane adsorbers for actinide separations in lieu of resin-based chromatography. Traditional resin columns rely on diffusion to drive target isotopes into the pores of the resin beads. At trace-level isotope concentrations, the diffusion driving force will be low, and the resin columns will require long residence times to achieve sufficient levels of extraction. Alternatively, an extractive membrane adsorber allows for isotopes to be conveyed to the binding sites by convective flow. For this application, the distribution of PVDF-g-EGMP along the pores of the membrane will improve the capacity for Pu and other actinides, and a bind-and-elute strategy can be developed for the sequential separation of each actinide from a mixed low-level source. A similar strategy can be adapted from elution procedures for commercial radiochromatography resins. Horwitz et al. developed an elution procedure for mixed actinides using TRU resin (Eichrom Technologies, Lisle, IL, USA) containing octyl(phenyl)-*N*,*N*-diisobutylcarbamoylmethylphosphine oxide (CMPO) in tri-*N*-butyl phosphate (TBP) [31]. The study demonstrated that tri-, tetra- and hexavalent actinides can be eluted sequentially from the resin using solutions composed of various concentrations of hydrochloric acid and other reagents. Other studies have demonstrated that EGMP-based copolymers can be regenerated over multiple bind-and-elution cycles for repeated use. In a study by Mhatre et al., thin films composed poly(bis[2-(methacryloyloxy)ethyl] phosphate) (poly(BMEP) produced from a divinyl ester form of EGMP) did not exhibit any reduction in Pu^4+^ recovery even after three desorption cycles with a solution composed of 1 M nitric acid and 0.2 M hydrazine hydrate and hydroxylamine [8]. For membranes containing PVDF-g-EGMP, it is likely that a similar desorption step can be performed to regenerate the membrane for multiple cycles.

## 4. Conclusions

An ATRP procedure was developed to graft PEGMP from PVDF, creating a graft copolymer capable of extracting Pu from water at neutral pH conditions. The copolymer can be added to PVDF dope solutions to form membranes by nonsolvent induced phase separation for Pu capture and alpha spectrometry. Permeability studies with membranes composed of 10 wt% PVDF-*g*-EGMP indicated that water permeability improved with the addition of the copolymer, and a wide range of permeabilities was achieved by adjusting casting conditions. The copolymer membranes exhibited ^238^Pu uptakes as high as 59.9 ± 3.0% and 19.3 ± 3.5% from DI water and synthetic seawater solutions that were passed through the membrane. SEM-EDS analysis showed that the graft copolymer was distributed throughout the depth of the membrane, leading to ^238^Pu peak tailing in the resulting alpha spectra. Future studies will investigate methods to increase the surface concentration of PVDF-*g*-EGMP within the membrane, including changes to the copolymer formulation and phase inversion procedure to promote surface migration during membrane formation.

## Figures and Tables

**Table 1:** Compositions of the dope solutions prepared for casting poly(vinylidene fluoride) (PVDF) membrane composed of 10% PVDF-*g*-EGMP (ethylene glycol methacrylate phosphate) in dimethylformamide (DMF).

	Membrane Dope Solutions			
	DMF	PVDF_534K_	PVDF-*g*-EGMP	Glycerol
Membrane	[g]	[g]	[g]	[g]
10% PVDF-*g*-EGMP No glycerol	47.2	7.5	0.83	0
10% PVDF-*g*-EGMP 2 wt% glycerol	46.1	7.5	0.83	1.11

## Data Availability

The data required to reproduce these findings are available by request to the corresponding author.

## Supplementary Materials

The following are available online at https://www.mdpi.com/article/10.3390/membranes12010003/s1, Table S1: Isotopic fractions of the stock 147 Bq/mL solution; Table S2: Ion content of the synthetic seawater used in the direct filtration ^238^Pu uptake studies; Table S3: Treatments for each batch of 10 wt% PVDF-*g*-EGMP membrane tested in the permeability study; Figure S1: Flux data for each 10 wt% PVDF-*g*-EGMP batch tested in the permeability study; Figure S2: Alpha spectrum of the 5 Bq ^238^Pu smear efficiency calibration sample; Figure S3: Alpha spectra of the 0.5 mL samples taken for the ^242^Pu uptake study; Figure S4: Sessile drop contact angle measurements of 100% PVDF membranes; Figure S5: Sessile drop contact angle measurements of PVDF membranes casted with 10% PVDF-*g*-EGMP.
