# Supplementary material for: Membranes for the Capture and Screening of Waterborne Plutonium Based on a Novel Pu-Extractive Copolymer Additive
Source: Membranes (Basel). 2021 Dec 21;12(1):3. doi: 10.3390/membranes12010003 (PMC8779412; doi:10.3390/membranes12010003)
Supplement: Supplementary file 1 [file membranes-12-00003-s001.zip › membranes-1504987-supplementary.pdf]

SUPPLEMENTARY MATERIAL

Membranes for the Capture and Screening of Waterborne Plutonium Based on a Novel Pu-Extractive Copolymer Additive

James C. Foster [1,2], Timothy A. DeVol [2,3] and Scott M. Husson [1,*]

[1] Department of Chemical and Biomolecular Engineering, Clemson University, 127 Earle Hall, Clemson, SC 29634, USA; jcf@clemson.edu
[2] Nuclear Environmental Engineering Sciences, Radioactive Waste Management Center, Clemson University, Clemson, SC 29625, USA; devol@clemson.edu
[3] Department of Environmental Engineering and Earth Sciences, Clemson University, 342 Computer Court, Anderson, SC 29625, USA
* Correspondence: shusson@clemson.edu

CONTENT

Table S1: Isotopic fractions of the stock 147 Bq/mL solution used in the study

Table S2: Ion content of the synthetic seawater used in the direct filtration ^{238}Pu uptake studies

Table S3: Treatments for each batch 10 wt.% PVDF-*g*-EGMP membrane tested in the permeability study

Figure S1: Flux data for each 10 wt.% PVDF-*g*-EGMP batch tested in permeability study

Figure S2: Alpha spectrum of the 5 Bq ^{238}Pu smear efficiency calibration sample

Figure S3: Alpha spectra of the 0.5 mL samples taken for the ^{242}Pu uptake study

Figure S4: Sessile drop contact angle measurements of 100% PVDF membranes

Figure S5: Sessile drop contact angle measurements of PVDF membranes casted with 10% PVDF-*g*-EGMP.

Table S1: Isotopic mass fractions of Pu isotopes present in the 147 Bq/mL ^{242}Pu stock solution.

Isotope	Mass Fraction [%]	Specific Activity [Ci/g]	Mode of Decay [-]	Eα [keV]
^{238}Pu	0.003	1.70E+01	alpha	5499 (71%), 5456 (29%)
^{239}Pu	0.005	6.20E-02	alpha	5157 (71%), 5144(17%), 5106 (12%)
^{240}Pu	0.022	2.30E-01	alpha	5168 (73%), 5124 (27%)
^{241}Pu	0.006	1.00E+02	beta	...
^{242}Pu	99.962	3.90E-03	alpha	4902 (77%), 4858 (23%)
^{244}Pu	0.002	1.80E-05	alpha	4589 (81%), 4546 (19%)

Table S2: Ion content of the Instant Ocean aquarium salt used to prepare Pu-bearing solutions for the synthetic seawater trials.

Ion	Instant Ocean [ppm]	Typical Seawater [ppm]	*Ion*	Instant Ocean [ppm]	Typical Seawater [ppm]
Chloride	19290	19353	*Chromium*	<0.015	<0.01
Sodium	10780	10781	*Cobalt*	<0.015	<0.01
Sulfate	2660	2712	*Copper*	<0.015	<0.01
Magnesium	1320	1284	*Nickel*	<0.015	<0.01
Calcium	400	523	*Selenium*	<0.015	<0.01
(Bi)Carbonate	200	126	*Vanadium*	<0.015	<0.02
Bromide	56	67	*Zinc*	<0.015	<0.01
Strontium	8.8	7.9	*Molybdenum*	<0.01	0.01
Boron	5.6	4.5	*Aluminum*	<0.006	<0.001
Fluoride	1	1.28	*Lead*	<0.005	<0.001
Lithium	0.3	0.173	*Arsenic*	<0.004	0.002
Iodide	0.24	0.06	*Cadmium*	<0.002	<0.001
Barium	<0.04	<0.014	*Nitrate*	0	1.8
Iron	<0.04	<0.001	*Phosphate*	0	0.2
Manganese	<0.025	<0.001			

Table S3: Tabulated list of treatments for each 10 wt.% PVDF-*g*-EGMP membrane batch used in the permeability study.

Sample ID:	Vacuum treatment	Percent Glycerol	Casting Time
#1	Yes	0 wt.%	5 s
#2	No	0 wt.%	5 s
#3	Yes	2 wt.%	5 s
#4	No	2 wt.%	5 s
#5	Yes	0 wt.%	30 s
#6	No	0 wt.%	30 s
#7	Yes	2 wt.%	30 s
#8	No	2 wt.%	30 s

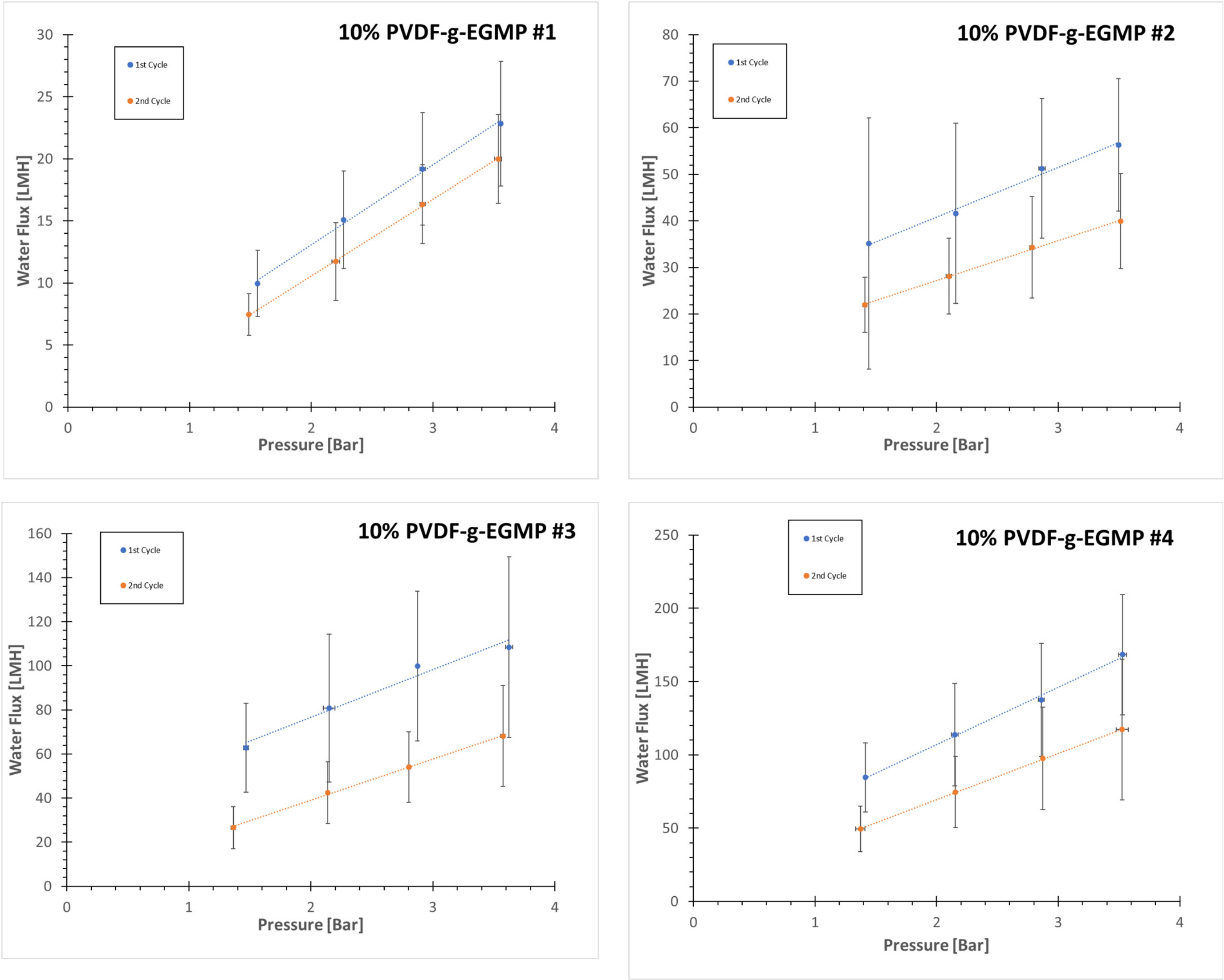

Figure S1: Resulting water flux data of the 10 wt.% PVDF-*g*-EGMP membranes from the permeability study.

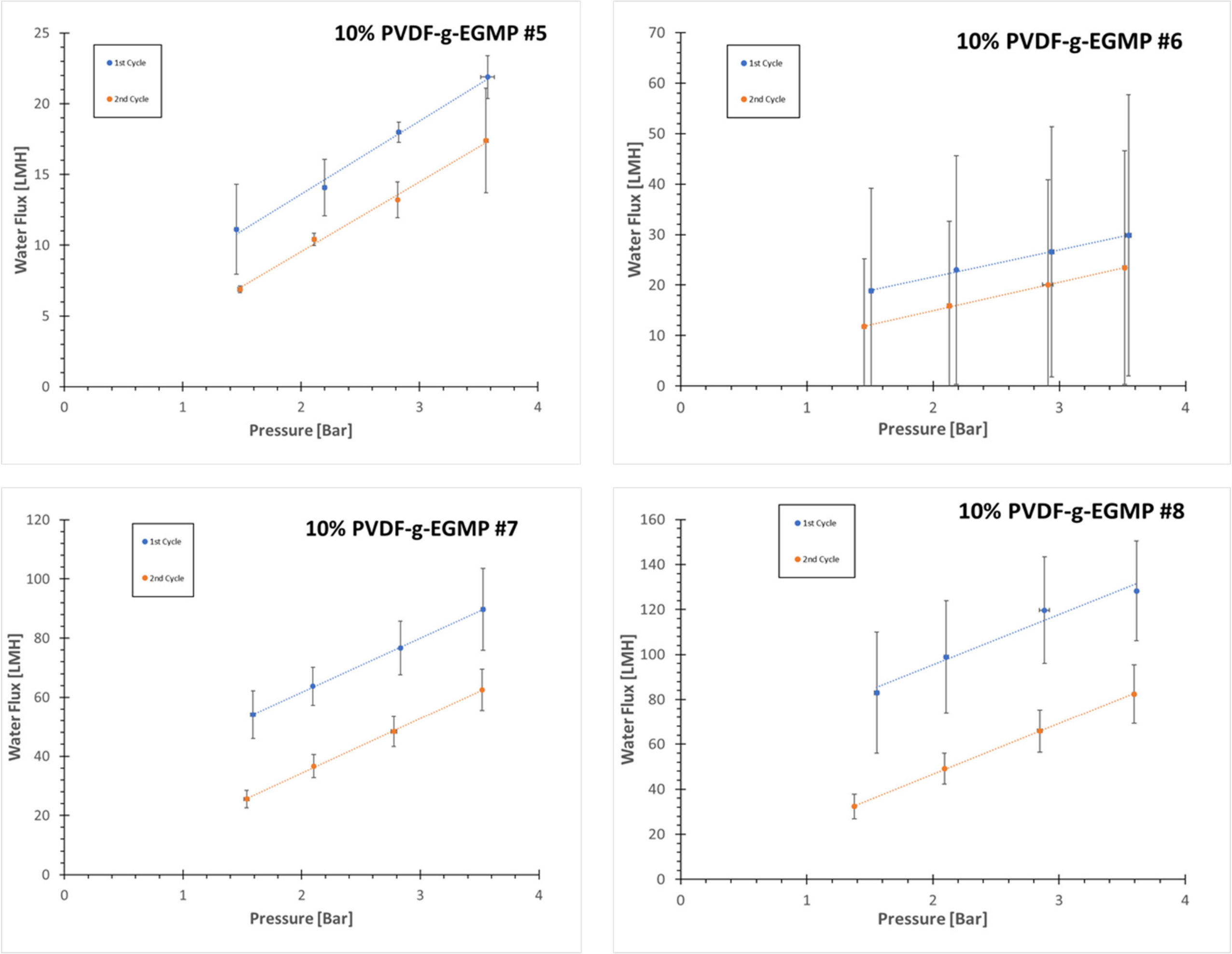

Figure S1 (Cont.): Resulting water flux data of the 10 wt.% PVDF-*g*-EGMP membranes from the permeability study.

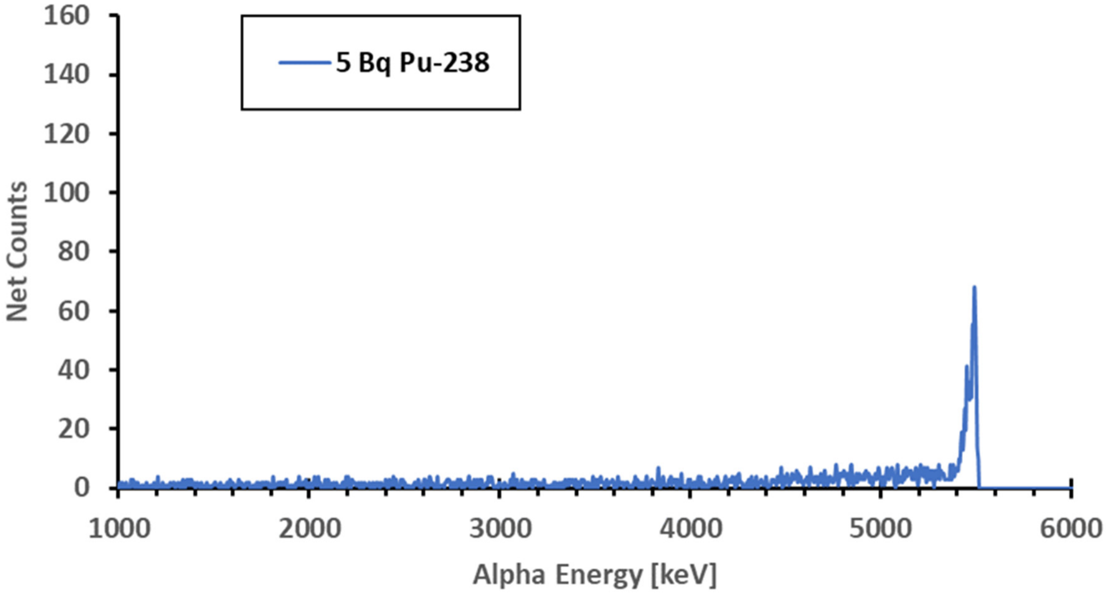

Figure S2: Alpha spectra of 5 Bq ^{238}Pu smeared over a 45 mm diameter PVDF membrane. The sample was dried and placed 5 mm from a 25 mm diameter PIPS detector and counted for 90 min, resulting in an absolute efficiency of 3.65% for the sample geometry.

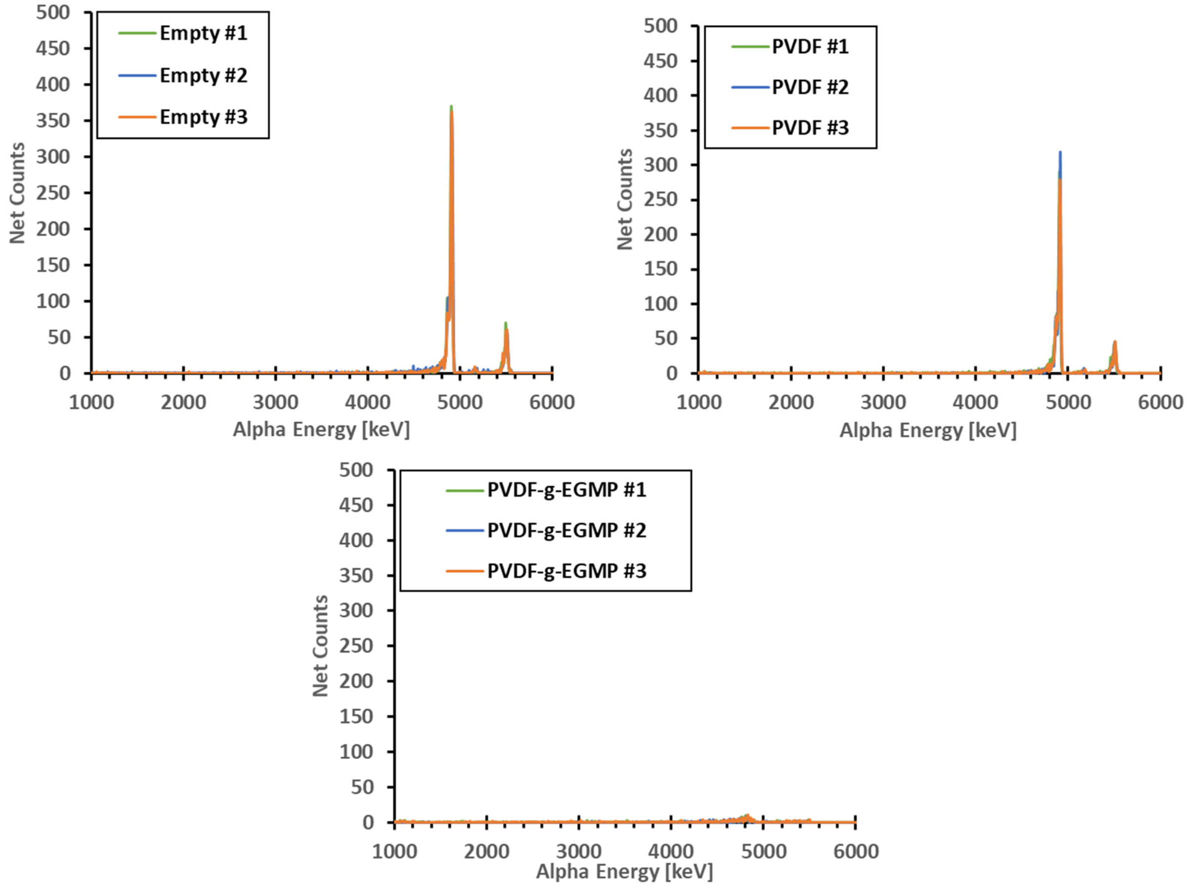

Figure S3: Resulting 5 h alpha spectra of the 0.5 mL ^{242}Pu solution samples collected after 24 h exposure to the (1) empty centrifuge vials; (2) 100 mg of PVDF resin; (3) 100 mg of PVDF-*g*-EGMP.

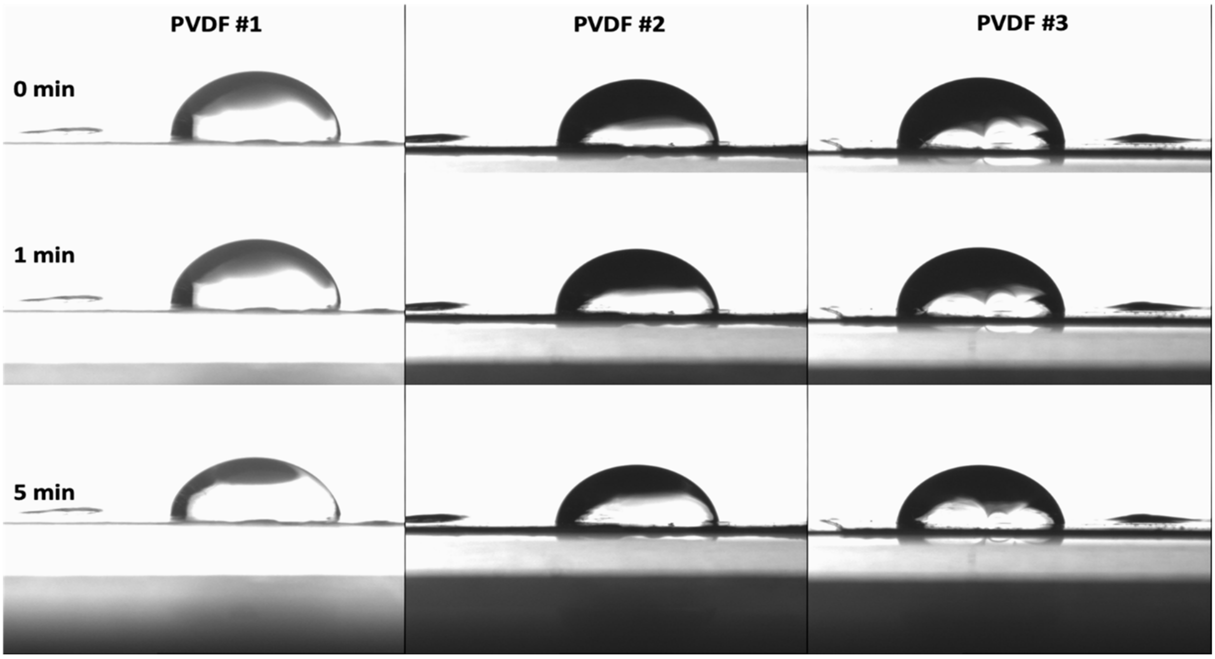

Figure S4: Contact angle measurements of 100% PVDF membranes using a 25µL droplet of DI water on the surface. Theta angle measurements (Θ) were measured at 0, 1, and 5 min intervals on three separate positions of the membrane.

**Sample** [-]	**Time** [min]	**Θ** [degree]
100% PVDF 1	0	88.6
…	1	88.6
…	5	85.3
100% PVDF 2	0	90.8
…	1	90.3
…	5	86.1
100% PVDF 3	0	91.7
…	1	90.3
…	5	85.5
**Time**	**Mean (Θ)**	**Std. Dev. (Θ)**
0 min	90.4	1.59
1 min	89.7	0.98
5 min	85.6	0.42

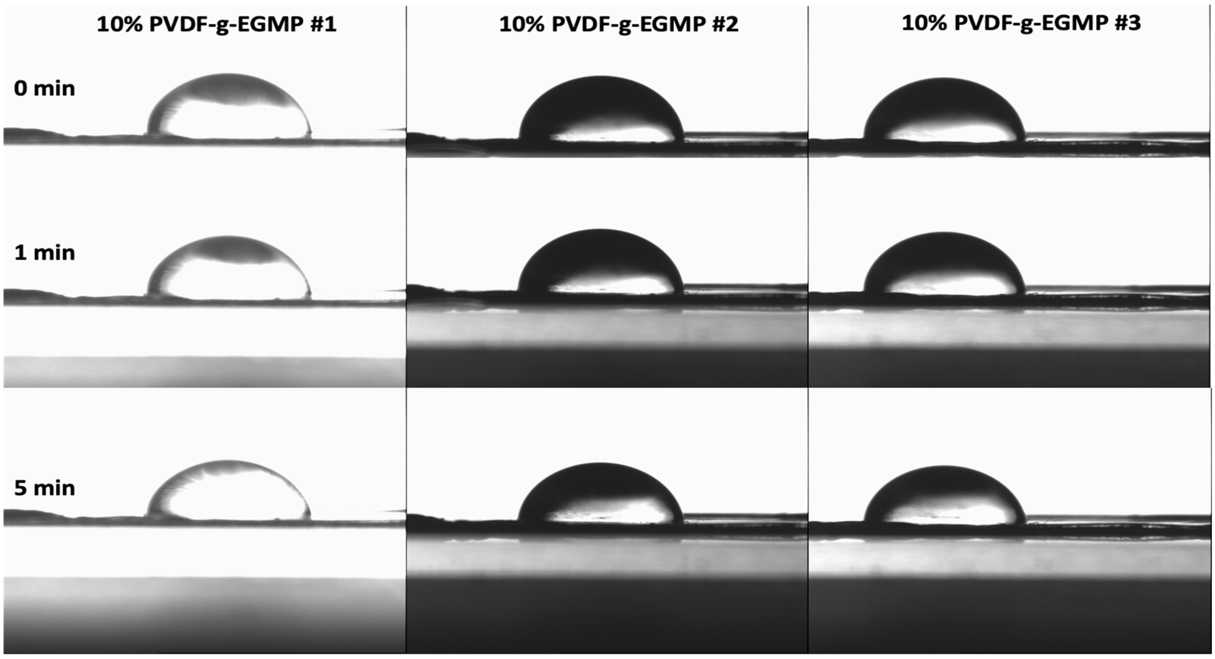

Figure S5: Contact angle measurements of PVDF membranes containing 10 wt.% PVDF-*g*-EGMP using a 25μL droplet of DI water on the surface. Theta angle measurements (Θ) were measured at 0, 1, and 5 min intervals on three separate positions of the membrane.

**Sample**<br>**[-]**	**Time**<br>**[min]**	**Θ**<br>**[degree]**
100% PVDF 1	0	85.6
…	1	85.1
…	5	81.8
100% PVDF 2	0	85.5
…	1	84.5
…	5	80.4
100% PVDF 3	0	84.6
…	1	83.7
…	5	79.6
**Time**	**Mean (Θ)**	**Std. Dev. (Θ)**
0 min	85.2	0.55
1 min	84.4	0.70
5 min	80.6	1.11
